# Cryptococcal pericarditis with unexplained lymphadenopathy in an immunocompetent patient: a case report

**DOI:** 10.3389/fmed.2025.1688934

**Published:** 2025-12-12

**Authors:** Jie Shu, Dongdong Zhou

**Affiliations:** General Department of the First Affiliated Hospital of Ningbo University, Ningbo, Zhejiang, China

**Keywords:** pericardial effusion, invasive fungal infections, *Cryptococcus* spp., multiple lymphadenopathy, cryptococcal lung disease

## Abstract

**Objective:**

To present a rare case of cryptococcal pericarditis with unexplained multiple lymphadenopathies in an immunocompetent patient.

**Background:**

Fungal pericarditis is an uncommon infection that may result from hematogenous dissemination, direct extension, or iatrogenic inoculation. Cryptococcal pericarditis typically occurs in immunocompromised hosts and is exceedingly rare in immunocompetent individuals. We describe a case of cryptococcal pericarditis with multiple lymphadenopathies in an immunocompetent patient.

**Case Report:**

A 40-year-old male with no underlying disease or history of high-risk behaviors presented with chronic cough and sputum production. Imaging revealed bilateral pulmonary lesions, widespread lymphadenopathy, and pericardial effusion. Serum and pericardial fluid cryptococcal antigen tests were positive. HIV testing (fourth-generation antigen/antibody ELISA) was negative, and immunologic evaluation was unremarkable. Pericardiocentesis drained 585 mL of effusion. The patient was treated with intravenous antifungal therapy with fluconazole (0.4 g daily) for 1 week, followed by oral fluconazole for 4 months. Follow-up echocardiography performed 2 weeks after discharge revealed no significant pericardial effusion, whereas a subsequent at 4 months after discharge demonstrated a small residual effusion. The patient's cough and sputum production had also improved. Throughout the hospitalization and post-discharge period, the patient was able to maintain normal physical activity without functional limitations.

**Conclusions:**

This report presents a rare case of cryptococcal pericarditis with unexplained multiple lymphadenopathies in an immunocompetent patient, highlighting that fungal infection should be considered even in immunocompetent hosts with pericarditis.

## Introduction

Fungal pericarditis is a rare but potentially fatal condition, with a reported mortality rate of up to 50% (1). The most common etiologic fungi are *Candida* and *Aspergillus* (2, 3). Among patients with fungal pericarditis, *Mucor* carries the highest reported mortality rate (88%), followed by *Aspergillus* (71%), *Cryptococcus* (33%), and *Candida* (24%) (4). *Cryptococcus* is a relatively rare cause of fungal pericarditis but carries a mortality rate second only to *Mucor* and *Aspergillus*.

Fungal infections are typically opportunistic. In humans, primary pulmonary infection with *Cryptococcus* occurs via inhalation, which may be asymptomatic. *Cryptococcus* causes moderate to severe disease in immunocompromised patients through hematogenous dissemination (5). Risk factors for severe fungal infections include Human immunodeficiency virus (HIV), autoimmune diseases, malignancy, transplantion, and underlying conditions such as chronic obstructive pulmonary disease (COPD), cirrhosis, and diabetes (6). In addition to the lungs, *Cryptococcus* often co-infects the central nervous system (7). Cryptococcal pneumonia complicated by pericarditis remains exceedingly rare.

This report describes a 40-year-old immunocompetent male with cryptococcal pericardial effusion and multiple lymphadenopathy.

## Case report

**Figure 1:**
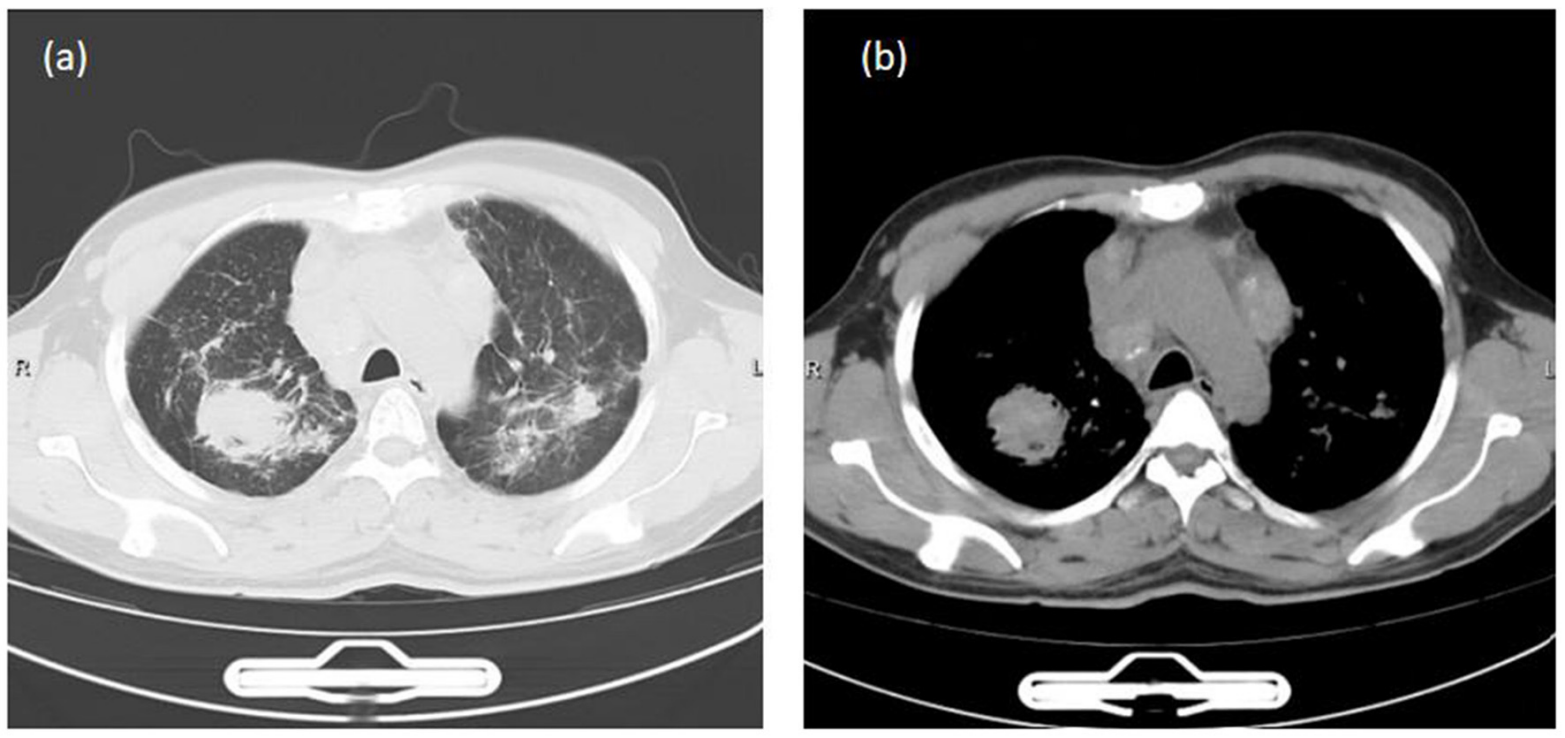
Chest computed tomography (CT) revealed multiple bilateral pulmonary lesions, multiple enlarged hilar and mediastinal lymph nodes, pericardial effusion, and lymphadenopathy in the cardiophrenic angle. **(a)** Lung window. **(b)** Mediastinal window.

A 40-year-old male presented to our outpatient clinic with a persistent cough and sputum production lasting over 1 month. The patient has no symptoms such as fever, weight loss, night sweats, chest pain, or dyspnea. Initial chest computed tomography (CT) revealed multiple bilateral pulmonary lesions, multiple enlarged hilar and mediastinal lymph nodes, pericardial effusion, and lymphadenopathy in the cardiophrenic angle (Figure 1). He had no history of hypertension, diabetes, smoking, alcohol consumption, or long-term corticosteroid or immunosuppressive therapy.

On admission, the patient's vital signs were as follows: temperature 36.8 °C, pulse 90 beats/min, respiratory rate 14 breaths/min, blood pressure 118/84 mmHg. The patient was alert and oriented, with palpable, enlarged lymph nodes in the bilateral supraclavicular, axillary, and inguinal regions. Breath sounds were coarse bilaterally, without obvious wheezes or crackles. Heart sounds were muffled, and no jugular venous distension was observed.

**Figure 2:**
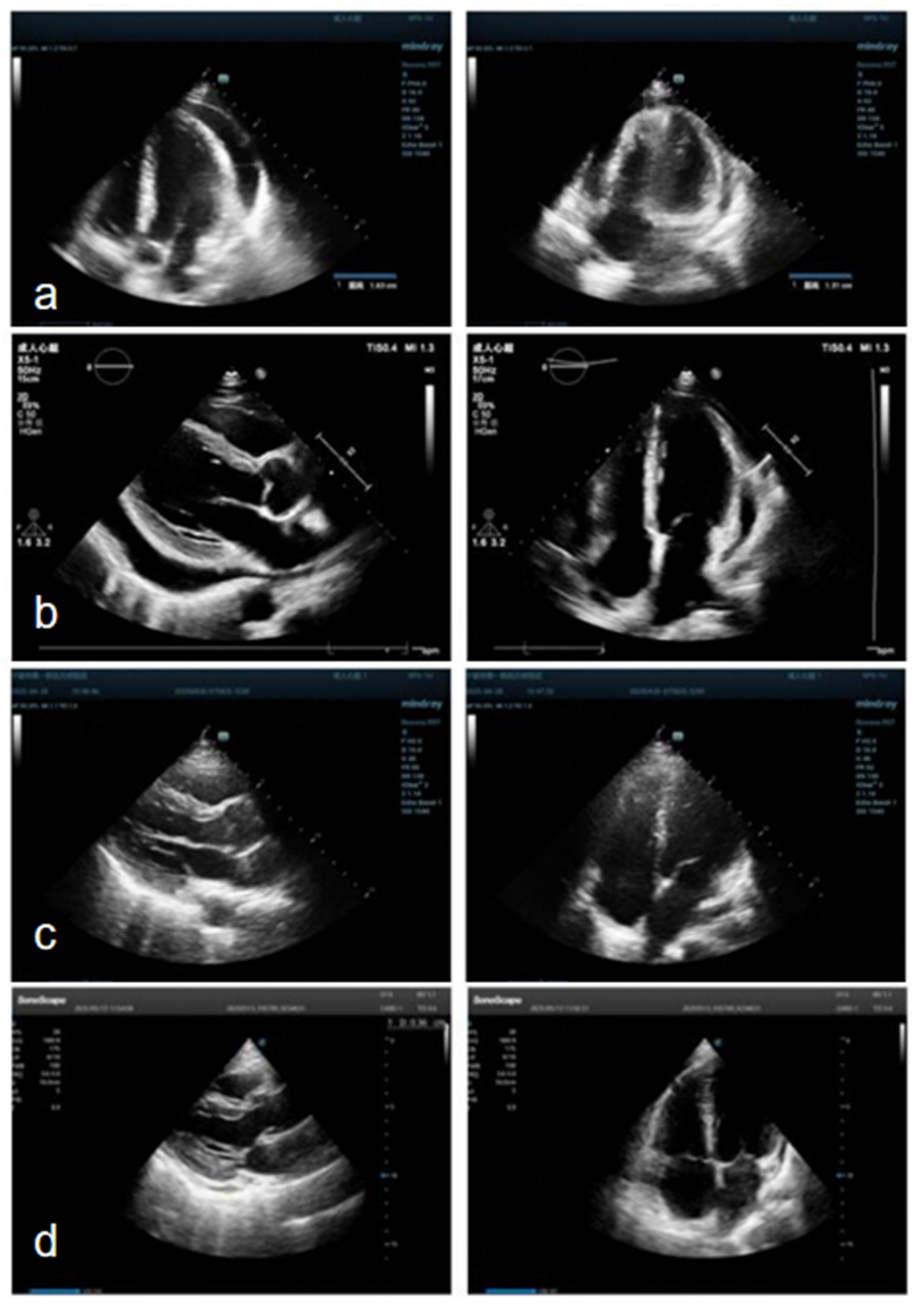
Echocardiography on admission revealed a moderate pericardial effusion along the left ventricular lateral wall, measuring 16 mm at its maximum diameter **(a)**. During hospitalization, repeat echocardiography demonstrated progression to a large posterior left ventricular effusion, measuring 21 mm at its maximum diameter **(b)**. Follow-up echocardiography performed 2 weeks after discharge showed complete resolution of the effusion **(c)**. At 4 months post-discharge, a small residual pericardial effusion measuring 4 mm was observed along the posterior left ventricular wall **(d)**.

**Figure 3:**
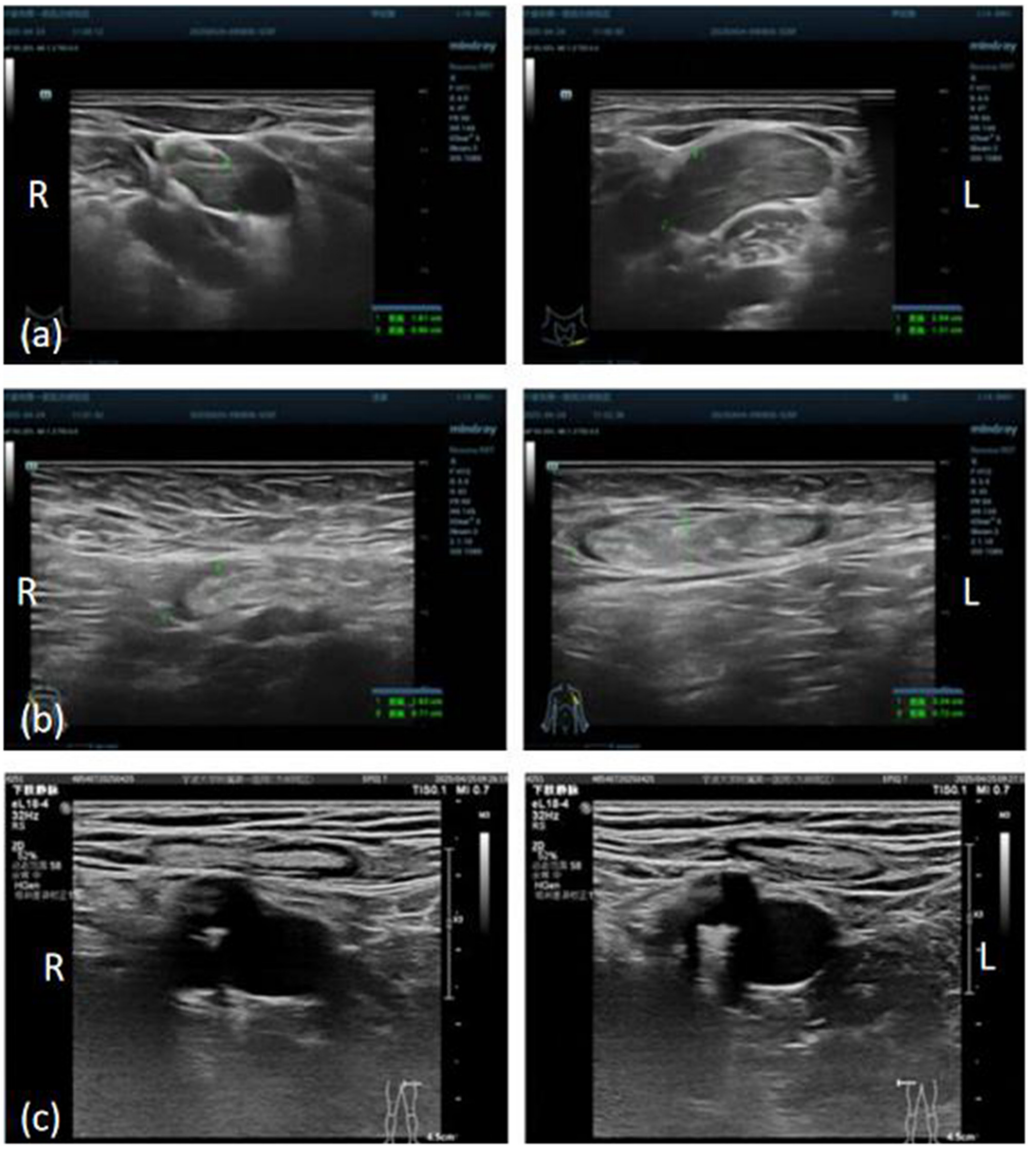
Ultrasound of superficial lymph nodes demonstrated multiple enlarged lymph nodes in the bilateral supraclavicular **(a)**, axillary **(b)**, and inguinal **(c)** regions.

Initial laboratory evaluation revealed a white blood cell count was 5.3 × 10^9^/L, with a decreased neutrophil percentage (33.8%), elevated lymphocytes (55.4%), and increased monocytes (10.2%). Serum CA125 was mildly elevated at 40.1 U/mL. Erythrocyte sedimentation rate (ESR) was 56 mm/h. Albumin was slightly decreased (38.5 g/L), while globulin was elevated (42.2 g/L). Lactate dehydrogenase (LDH) and LDH isoenzymes were also elevated at 301 U/L and 106 U/L, respectively. The serum cryptococcal antigen test returned a positive result. C-reactive protein, procalcitonin, B-type natriuretic peptide (BNP), liver enzymes, renal function tests, hemoglobin, tuberculosis antibodies, tumor markers, uantitative immunoglobulin levels (IgG, IgA, IgM) and autoimmune screening, including ANA and ENA panels, were all within normal limits. A panel of 13 respiratory pathogens and HIV testing with the fourth-generation antigen/antibody ELISA yielded negative results. The patient also denied any history of high-risk behaviors associated with HIV transmission. Transthoracic echocardiography at admission demonstrated a moderate pericardial effusion, with the maximal diameter of 16 mm located along the lateral wall of the left ventricle (Figure 2a). Ultrasound of superficial lymph nodes demonstrated multiple enlarged lymph nodes in the bilateral supraclavicular, axillary, and inguinal regions (Figure 3).

**Figure 4:**
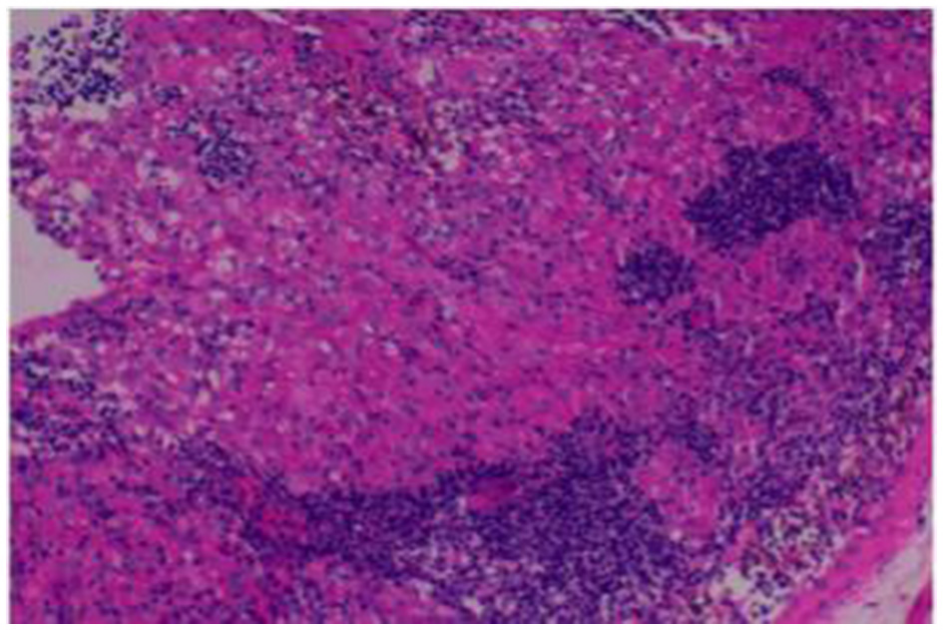
Histopathological examination of the left supraclavicular lymph node revealed non-caseating granulomatous inflammation.

Considering the inverted white blood cell ratio and multiple lymph node enlargement, hematological malignancies cannot be ruled out. M protein, blood and urine light chains, and immunoglobulin G subtypes were further tested. The results showed that all the above indicators were negative. Given the combination of pulmonary infection, pericardial effusion, and elevated ESR, tuberculosis should be considered. Tuberculosis T-cell testing and sputum acid-fast bacilli smears were performed, both of which returned negative results. A markedly enlarged left supraclavicular lymph node was selected for biopsy, and histopathological examination revealed granulomatous inflammation (Figure 4). Further fungal staining was negative.

During hospitalization, repeat echocardiography revealed a large pericardial effusion, with a maximal diameter of 21 mm along the posterior wall of the left ventricle (Figure 2b). On day 3 of hospitalization, pericardial puncture and drainage were performed, yielding 360 mL on the first day, 175 mL on the second day, and 50 mL on the third day, for a total of 585 mL. Follow-up echocardiography on day 6 of hospitalization, showed no residual effusion, and the pericardial drainage tube was subsequently removed.

Pericardial fluid was sent for routine analysis, biochemical testing, Mycobacterium tuberculosis DNA detection (Xpert), cryptococcal antigen testing, and bacterial and fungal cultures. Analysis revealed an exudative effusion characterized by lymphocyte predominance, total protein of 67.3 g/L, lactate dehydrogenase of 870 U/L, glucose of 2.41 mmol/L, and adenosine deaminase of 57.5 U/L. Both bacterial and fungal cultures were negative. However, pericardial fluid cryptococcal antigen testing was positive, while nested real-time quantitative PCR for Mycobacterium tuberculosis was negative.

Based on these findings, cryptococcal pericarditis was diagnosed. Further occupational history revealed that water used for marble cutting was sourced from a nearby shrimp pond rich in organic matter, raising the suspicion that chronic inhalation of cryptococcal spores from this environment may have led to pulmonary infection and subsequent pericardial involvement.

In the absence of meningitis (e.g., headache, nausea, vomiting) and with a normal neurologic examination, lumbar puncture was not performed, especially considering the patient's limited financial resources. The patient received intravenous antifungal therapy with fluconazole (0.4 g daily) for 1 week, during which liver function tests remained within normal limits. At the patient's request for early discharge, treatment was transitioned to oral fluconazole 400 mg daily, which was continued for 4 months.

During outpatient follow-up, echocardiography performed 2 weeks after discharge showed complete resolution of the pericardial effusion (Figure 2c). A subsequent echocardiogram at 4 months demonstrated a small residual effusion (maximum diameter 4 mm) along the posterior wall of the left ventricle (Figure 2d). The patient's respiratory symptoms, including cough and sputum production, also improved. Throughout hospitalization and follow-up, the patient remained physically active without restrictions in daily activities.

## Discussion

This case highlights the importance of considering fungal infections, including cryptococcosis, in the differential diagnosis of pericardial effusion even in immunocompetent individuals. In addition to pericardial effusion caused by viruses, bacteria, and tuberculosis, clinicians should be more vigilant against fungal pericarditis. Early diagnosis and early treatment are extremely important for fungal pericarditis.

*Cryptococcus* is ubiquitous in the environment, particularly in soil enriched with organic matter, decaying vegetation, and bird droppings (8–12). *Cryptococcus* comprises two major pathogenic species: *Cryptococcus neoformans* and *Cryptococcus gattii*. Cryptococcosis is a globally distributed invasive fungal disease, typically acquired through the inhalation of environmental spores or yeast cells (13). The infection typically initiates in the lungs (14) and may lead to pneumonia in immunocompromised individuals. In immunocompetent hosts, however, the organism is often cleared by the immune system or remains latent and asymptomatic. Once inhaled, *Cryptococcus* can evade phagocytic killing through various virulence mechanisms, including capsule formation, melanin production, and urease secretion, allowing it to survive and replicate within the host (15). If host immunity is later compromised, latent cryptococcal infection may disseminate hematogenously, most commonly involving the central nervous system. Without prompt and effective treatment, cryptococcal meningoencephalitis can carry a mortality rate of up to 90% (13).

Previous studies have demonstrated that pulmonary fungal infections predominantly occur in patients with HIV or other forms of immunosuppression. However, increasing evidence suggests that individuals without classic risk factors or overt immunosuppression may also develop pulmonary fungal infections (16). *Cryptococcus* is capable of disseminating to virtually any organ system (17). Although the lungs, brain, and skin are commonly involved, involvement of peripheral lymph nodes and the pericardium is rare (18). A previous case report described a 26-year-old immunocompetent male with disseminated cryptococcosis presenting with mediastinal and hilar lymphadenopathy (19). Another report documented an 11-year-old girl with disseminated cryptococcosis involving the lungs, generalized lymphadenopathy, and hepatosplenomegaly (18). In contrast, the present case was notable for predominant pericardial involvement with unexplained generalized lymphadenopathy in an immunocompetent male, highlighting its unique clinical presentation. Given that the patient was immunocompetent, exhibited no neurological signs or symptoms suggestive of meningeal involvement, and considering the patient's limited financial resources, a lumbar puncture was not performed after a shared decision (20).

Cryptococcal antigen testing is a rapid and sensitive method widely used in serum and cerebrospinal fluid. In patients without disseminated disease, serum from those with pulmonary cryptococcosis rarely tests positive (21). In this case, although cultures of the pericardial effusion were negative for bacteria and fungi, cryptococcal antigen was detected in both the pericardial effusion and serum. We acknowledge the limitations of pericardial antigen testing, and the sensitivity and specificity of cryptococcal antigen positivity in pericardial effusion remain uncertain, likely due to the rarity of cryptococcal pericarditis and limited available research. Nonetheless, given the high sensitivity and specificity of cryptococcal antigen testing in serum and cerebrospinal fluid, cryptococcal pericarditis was considered the primary diagnosis in this patient.

Additionally, adenosine deaminase (ADA) levels have been reported in six cases of cryptococcal pleural effusions, with approximately half showing elevated levels. Detection of cryptococcal antigen in pleural fluid is an important diagnostic tool for early identification of the infection. Even in cases with elevated ADA, cryptococcal infection should be considered in patients with unexplained exudative effusions (22). In this patient, ADA levels in the pericardial effusion were elevated; however, PCR testing for Mycobacterium tuberculosis was negative, making tuberculous pericardial effusion unlikely.

Pericardial effusion is a relatively common clinical condition with diverse manifestations and etiologies. It may result from autoimmune diseases, malignancies, metabolic disorders, or infections (23). Clinical presentation ranges from asymptomatic cases to life-threatening conditions, primarily depending on the rate of fluid accumulation and the underlying cause. Notably, a large pericardial effusion does not necessarily indicate cardiac tamponade, as chronic large effusions may be well tolerated (24, 25). In the present case, the patient was a young adult male with strong compensatory capacity and a non-acute pericardial effusion. Although echocardiography revealed a maximum posterior left ventricular wall effusion of 21 mm, corresponding to a large volume, the patient remained asymptomatic with normal motor function.

Occupational and environmental exposures are often linked to fungal infections. In this case, the patient had long-term occupational exposure to water sourced from a shrimp pond during marble cutting. Cryptococcal spores are abundant in organic-rich pond soil, where elevated humidity can significantly enhance activity of the spores (5, 26–28). This environmental exposure may have served as the primary route of cryptococcal infection in this patient. No environmental sampling was performed in this case. Any association between the patient's occupational or environmental exposures and the infection is inferred from previously published environmental survey studies. However, the mechanism underlying cryptococcal pericarditis with unexplained lymphadenopathy in this apparently immunocompetent patient remains unclear.

This case has several limitations. First, a lumbar puncture was not performed. Although the patient exhibited no neurological signs, subclinical central nervous system involvement cannot be completely excluded. Given the patient's limited financial resources, the decision to forego lumbar puncture was made after discussion with the patient. Secondly, although fungal staining of the lymph node biopsy was negative, this may be related to a low cryptococcal burden within the nodes. In light of the two previously reported cases of disseminated cryptococcosis presenting with lymphadenopathy, cryptococcal involvement cannot be entirely excluded. However, given the absence of histopathological confirmation, attributing the lymphadenopathy to cryptococcosis remains uncertain. Additionally, rare conditions such as idiopathic CD4 lymphocytopenia (29) and inborn errors of immunity, including GATA2 deficiency (30), have been reported as potential underlying causes of cryptococcosis in non-HIV patients. In this case, routine immune function tests were normal, but the presence of such rare disorders cannot be entirely ruled out.

Currently, there is no universally established diagnostic or therapeutic protocol for cryptococcal infection. According to current clinical guidelines, the optimal fungicidal induction regimen for disseminated cryptococcosis consists of liposomal amphotericin B combined with flucytosine. In resource-limited settings, a single high dose of liposomal amphotericin B along with flucytosine and fluconazole is commonly administered. The induction phase typically lasts at least 2 weeks. During the consolidation phase, fluconazole at a dosage of 400–800 mg/day is recommended for 8 weeks, followed by a maintenance dose of 200 mg/day for 12 months. This patient was treated with fluconazole monotherapy. While fluconazole offers favorable pharmacokinetics, this approach deviates from guideline-recommended induction therapy and may increase the risk of relapse or resistance. Therefore, long-term monitoring is essential. In this case, although the patient responded well to treatment, showed a favorable prognosis, and remained under regular outpatient follow-up after discharge, the limitations of fluconazole monotherapy must be acknowledged.

## Conclusion

We report a rare case of cryptococcal pericarditis with multiple lymphadenopathies in an immunocompetent patient. Cryptococcal pericarditis is rare in immunocompetent patients. The primary route of infection for cryptococcal infection is through the pulmonary system; atypical manifestations of fungal infection include hematogenous spread leading to cryptococcal pericarditis. Clinicians should expand their differential diagnoses to include cryptococcosis even in patients without overt immunosuppression so as to identify and intervene early. Our case highlights the rarity and complexity of cryptococcal pericarditis. Given its high mortality, cryptococcal pericarditis demands early recognition, accurate diagnosis, and prompt multidisciplinary management.

## Data Availability

The raw data supporting the conclusions of this article will be made available by the authors, without undue reservation.
